# Is routine ophthalmoscopy really necessary in candidemic patients?

**DOI:** 10.1371/journal.pone.0183485

**Published:** 2017-10-24

**Authors:** Antonio Vena, Patricia Muñoz, Belen Padilla, Maricela Valerio, Maria Isabel Sanchez, Mireia Puig-Asensio, Jesus Fortun, Mario Fernandez-Ruiz, Paloma Merino, Juan Emilio Losa, Ana Loza, Rosa Ana Rivas, Emilio Bouza, for the CANDIPOP Project, GEIH-GEMICOMED (SEIMC), and REIPI

**Affiliations:** 1 Clinical Microbiology and Infectious Disease Division, Hospital General Universitario Gregorio Marañón, Madrid, Spain; 2 Instituto de Investigación Sanitaria Hospital Gregorio Marañón, Madrid, Spain; 3 Medicine Department, School of Medicine, Universidad Complutense de Madrid, Madrid, Spain; 4 Clinica Malattie Infettive AOU Santa Maria della Misericordia Piazzale Santa Maria della Misericordia, 15, Udine, Italy; 5 CIBER Enfermedades Respiratorias- CIBERES (CB06/06/0058), Madrid Spain; 6 Hospital Majadahonda, Madrid, Spain; 7 Hospital Universitario Vall D’ Hebron, Barcelona, Spain; 8 Hospital Ramón y Cajal, Madrid, Spain; 9 Hospital Universitario 12 de Octubre, Madrid, Spain; 10 Hospital Clínico, Madrid, Spain; 11 Hospital de Alcorcón, Madrid, Spain; 12 Hospital Universitario Virgen de Valme, Sevilla, Spain; 13 Hospital de Galdakano, Bilbao, Spain; Louisiana State University, UNITED STATES

## Abstract

The purpose of this study was to determine among patients with candidemia the real rate of ophthalmoscopy and the impact of performing ocular assessment on the outcome of the disease. We performed a post hoc analysis of a prospective, multicenter, population-based candidemia surveillance program implemented in Spain during 2010–2011 (CANDIPOP). Ophthalmoscopy was performed in only 168 of the 365 patients with candidemia (46%). Ocular lesions related to candidemia were found in only 13/168 patients (7.7%), of whom 1 reported ocular symptoms (incidence of symptomatic disease in the whole population, 0.27% [1/365]). Ophthalmological findings led to a change in antifungal therapy in only 5.9% of cases (10/168), and performance of the test was not related to a better outcome. Ocular candidiasis was not associated with a worse outcome and progressed favorably in all but 1 evaluable patient, who did not experience vision loss. The low frequency of ophthalmoscopy and ocular involvement and the asymptomatic nature of ocular candidiasis, with a favorable outcome in almost all cases, lead us to reconsider the need for systematic ophthalmoscopy in all candidemic patients.

## Introduction

Ocular involvement in patients with candidemia is classically reported as a significant complication with devastating consequences, a high rate of vision loss, and a potentially fulminant course [1–5]. Several guidelines consistently recommend routine ophthalmoscopy in all candidemic patients [6–9]. That is because when eye involvement is detected, antifungal therapy should include azoles or liposomal amphotericin B, occasionally combined with vitrectomy and/or intraocular antifungal drugs, and the duration of treatment should be extended for 4–6 weeks [6–9].

Nevertheless, recent series showed a low frequency of symptomatic ocular disease and a favorable clinical outcome in almost all patients with *Candida* eye involvement who received systemic antifungal treatment [10]. Moreover, the recommendation to perform systematic ophthalmoscopy is based not on the findings of a randomized controlled trial, but rather on the results of old, small-scale studies that do not clearly state clinical benefits [1–5].

Based on data from a recent population-based study of candidemia in Spain (CANDIPOP study), we determined the frequency of ophthalmoscopy and analyzed the impact of the examination on outcome.

## Materials and methods

### Study setting

The findings reported here are from a post hoc sub-analysis of the Population Study on Candidemia (CANDIPOP), a prospective, multicenter, population-based candidemia surveillance program implemented in 29 hospitals in Spain. The inclusion criteria, study population, main definitions, microbiological studies, and outcomes have been extensively described elsewhere [11]. Briefly, during the study period local laboratories daily identified patients and reported them to study coordinators, who collected data using a standardized case report form. Data included demographic and clinical characteristics, risk factors for candidemia, antifungal management and source control. Thirty-day follow-up outcome was recorded for each patient. Given the observational nature of the study, patients were managed according to routine clinical care.

Severity of the infection and Pitt bacteremia score were recorded on the day of blood culture sampling [12]. Proven catheter-related candidemia was defined according to current guidelines [13], whereas secondary candidemias required the microbiological documentation of the same *Candida* species at the origin of infection [11]. When there was no apparent infection at another site, candidemia was classified as primary. An episode of candidemia was defined as persistent when patients had positive follow-up blood cultures performed according to IDSA guidelines [9].

The local institutional review boards of each participating center approved this study, and written informed consent was obtained from each patient before enrollment (IRB of the coordinating center for this study: Comité Ético de Investigación Clínica, Hospital General Universitario Gregorio Marañón).

### Populations

Systematic dilated ophthalmoscopy was recommended at baseline for all patients included in the study, although only pathological findings suggestive of *Candida* ocular involvement were recorded in the general CANDIPOP database. To overcome this limitation, we asked the 29 participating hospitals to re-check whether ophthalmoscopy had been performed in patients aged >16 years who had experienced an episode of candidemia. Eleven hospitals complied with our request.

Therefore, we included the 365 episodes of candidemia from the CANDIPOP study for which information was available on whether or not ophthalmoscopy had been performed.

### Collection of ophthalmologic data

The study coordinators were invited to retrospectively review the results of all ophthalmological examinations in order to categorize ocular involvement as proven, probable, or possible *Candida* chorioretinitis or endophthalmitis [10]. To be considered proven, ocular candidiasis had to be diagnosed based on the presence of an ocular lesion and on isolation of the microorganism from the vitreous humor by culture or histopathology-based identification. Probable *Candida* endophthalmitis consisted of vitritis or fluffy lesions with extension into the vitreous humor. Probable *Candida* chorioretinitis included deep focal white infiltrates in the retina, hemorrhages, Roth spots, or cotton wool spots. In patients with diabetes, hypertension, or concomitant bacteremia, ocular involvement was classified as possible based on previous criteria [10]. Injection of intravitreal antifungal agents or intravitreal corticosteroids was recorded, as was the need for vitrectomy.

The outcome of ocular candidiasis was considered successful when follow-up ophthalmoscopy revealed resolution of the retinal lesion or of active inflammation.

### Statistical analysis

Categorical variables are presented as absolute numbers and their relative frequencies. Quantitative variables are presented as means and standard deviation (SD) if normally distributed and as median and interquartile range (IQR) if non-normally distributed. We compared categorical variables between groups using the Pearson chi-square and Fisher exact tests; we compared continuous variables using the Mann-Whitney test or a two-tailed *t* test.

Risk factors for 30-day mortality were analyzed using the Cox regression model assuming proportional hazards.

One of our main objectives was to analyze the impact of systematic ophthalmoscopy on the outcome of patients with candidemia. Since very early mortality is generally associated with the impossibility to perform an ophthalmoscopy, patients who died within 3 days after withdrawal of blood cultures (12 patients) were excluded from mortality analysis to rule out potential bias.

Associations are given as odds ratio (OR) with the 95% confidence interval (95% CI). Data were analyzed using SPSS Statistics for Windows, Version 17.0 (SPSS Inc., Chicago, Illinois, USA). Statistical significance was established at *p* < 0.05.

## Results

### Frequency of ophthalmoscopy and incidence of ocular candidiasis

Ophthalmoscopy was performed in only 168 of the 365 patients with candidemia (46%). Abnormalities suggestive of *Candida* eye involvement were present in only 13/168 cases (7.7%).

Probable *Candida* endophthalmitis occurred in 2 patients, whereas probable and possible chorioretinitis were present in 8 and 3 cases, respectively. Interestingly, all patients but 1 (a patient with probable bilateral chorioretinitis who had low visual acuity) were asymptomatic when the diagnosis of ocular candidiasis was established. Accordingly, symptomatic ocular disease due to *Candida* occurred in 0.27% of the whole candidemic population (1 out of 365 patients) and in 7.6% of the 13 patients with ocular involvement (1 out of 13 patients).

### Assessment of performing or not ophthalmoscopy on 30-day mortality

Overall, 102/365 patients (27.9%) died within 30 days of the episode; 12 of these patients died within 3 days of blood sample collection and were, therefore, excluded from the mortality analysis. Univariate analysis of risk factors for 30-day mortality in the 90 remaining patients showed that the factors associated with poor outcome were admission to the ICU, previous renal disease, HIV infection, previous corticosteroid treatment, primary source of infection, septic shock, higher Pitt score, and need for hemodialysis as a complication of candidemia. The factors associated with a better outcome were admission to a surgical ward, receipt of azoles, and ophthalmoscopy.

However, when a multivariate analysis was performed, the independent risk factors for mortality **(Table 1)** were septic shock at presentation of candidemia, primary candidemia, and a high Pitt score. Performance of ophthalmoscopy did not remain an independent protective factor for 30-day mortality (OR, 0.59; 95% CI, 0.34–1.05; *p* = 0.08).

**Table 1:** Multivariate logistic regression analysis of prognostic factors associated with 30-day mortality.

VARIABLE	OR	95% CI	p-*value*
**Septic shock**	**2.55**	**1.03–6.34**	**0.04**
**Primary candidemia**	**1.8**	**1.04–3.22**	**0.03**
**Pitt score**	**1.20**	**1.05–1.37**	**<0.01**
Performance of ophthalmoscopy	0.59	0.34–1.05	0.08
Corticosteroids therapy within previous 30 days	0.58	0.33–1.02	0.06
Surgical ward	0.51	0.25–1.03	0.06
HIV/AIDS	0.49	0.11–1.8	0.26
Mucositis	0.37	0.13–1.01	0.06

### Clinical impact and outcome of ocular candidiasis

Antifungal therapy was changed after dilated ophthalmoscopy in 10/168 patients (5.9%). Six patients required a change in the class of antifungal administered, whereas 4 patients were prescribed extended treatment.

The outcome of ocular candidiasis is summarized in **Table 2**. Overall, information on the evolution of ocular candidiasis was available in 7/13 patients (53.8%), since 5 died and fundoscopy follow-up was not available in the remaining patient. Antifungal treatment was considered successful in 6/7 patients. None of the patients with *Candida* eye involvement needed intravitreal injection of antifungals or surgery.

**Table 2:** Clinical characteristic of patients with endogenous ocular candidiasis.

AGE (y)/sex	Risk factor	Results of ophthalmological examination	Other organs involvement	Time to funduscopic examination	Antifungal therapy before fundoscopy	Treatment	Days between diagnosis and first follow-up fundoscopy	Outcome
68/M	Surgery for colon cancer. Broad-spectrum antibiotics. TPN*C*. *albicans* fungemia	Probable bilateral chorioretinitis	No	5 days	Fluconazole (No change in AF)	Fluconazole (6 weeks)	7 days	Complete resolution
55/M	Intra-abdominal aneurysm repair. Broad-spectrum antibiotics. Systemic corticosteroids. Persistent *C*. *albicans* fungemia requiring need for dialysis	Probable bilateral chorioretinitis	No	5 days	Fluconazole (3 days)	Micafungin IV (4 weeks)*	8 days	Complete resolution
45/M	TPN. Broad-spectrum antibiotics. Systemic corticosteroids. Persistent *C*.*albicans* fungemia requiring need for dialysis	Probable bilateral chorioretinitis	No	2 days	Anidulafungin (5 days)	Fluconazole (4 days)	Follow-up not available.	Not evaluable. Patient died
71/M	Abdominal surgery for colon cancer. TPN. Broad-spectrum antibiotics. Persistent *C*. *albicans* fungemia.	Possible bilateral chorioretinitis	Spleen involvement	5 days	Caspofungin (3 days)	Fluconazole (16 days) followed by caspofungin (23 days)	9 days	Complete resolution
17/F	Leukemia. Neutropenia. Broad-spectrum antibiotics. Persistent *C*.*guilliermondii* fungemia.	Probable bilateral chorioretinitis	Skin	3 days	Voriconazole (15 days) plus L-AMB (for the first 5 days)	Fluconazole plus micafungin (6 weeks)	16 days	Initial response. Persistence of ocular lesion in right eye at 2 weeks.
53/M	Cancer patients (cholangiocarcinoma) receiving systemic chemotherapy. Persistent *C*.*parapsilosis* fungemia.	Possible bilateral chorioretinitis	No	3 days	Fluconazole (No change in AF)	Fluconazole (32 days)	9 days	Complete resolution
62/M	Tongue cancer managed with chemotherapy. Broad-spectrum antibiotic therapy. TPN. Systemic corticosteroid therapy *C*. *parapsilosis* fungemia	Probable bilateral endophthalmitis	No	3 days	Caspofungin (2 days)	Fluconazole (4 days)	Follow-up not available.	Not evaluable. Patient died
43/F	Systemic corticosteroids. Broad-spectrum antibiotics. TPN. Persistent *C*. *albicans* fungemia requiring need for dialysis	Possible bilateral chorioretinitis	Yes. Septic thrombophlebitis	16 days	Fluconazole (No change in AF)	Fluconazole (20 days)	Follow-up not available.	Not evaluable
52/M	Broad-spectrum antibiotics. Brain tumor. *C*. *albicans* fungemia	Probable chorioretinitisin right eye	No	5 days	Fluconazole (No change in AF)	Fluconazole (5 weeks)	12 days	Complete resolution
76/M	Broad-spectrum antibiotics. Cancer. TPN. Persistent *C*. *albicans* fungemia requiring need for dialysis	Probable endophthalmitis in right eye and chorioretinitis in left eye	No	6 days	Fluconazole (No change in AF)	Fluconazole (11 days)	Follow-up not available	Not evaluable. Patient died
35 /F	Broad-spectrum antibiotics. SLE. Corticosteroids. ARF. Persistent *C*. *albicans* fungemia	Proven bilateral endophthalmitis	Brain	50 days	Fluconazole (2 days)	L- AMB and 5-flucytosine (8 weeks)	12 days	Ocular lesions persisted during follow-up but no active inflammation was detected
66 /M	Cancer. CRF. Previous antibiotics. Abdominal surgery. *C*. *albicans* fungemia	Probable chorioretinitis in both eyes	Spleen involvement	2 days	L-AMB (5 days)	Fluconazole (6 weeks)	Follow-up not available	Not evaluable
65 /M	Abdominal surgery for cancer. Broad-spectrum antibiotics. TPN. *C*. *albicans* fungemia	Probable bilateralchorioretinitis	No	6 days	Fluconazole (2 days)	Caspofungin (14 days)	Follow-up not available.	Not evaluable. Patient died

**ARF** acute renal failure; **AF** antifungal therapy **CRF** chronic renal failure; **L-AMB** liposomal amphotericin B; **SLE** systemic erythematosus lupus; **TPN** total parenteral nutrition.

*Patient number 2 was a renal transplant recipient who developed *C*. *albicans* septic shock after aortic aneurysm repair. Fungal infection was complicated with acute renal failure needing continuous renal replacement therapy andwith ocular candidiasis. Micafungin was administered because of stable levels with hemofiltration, lack of drug-drug interaction and because of its activity on *Candida* biofilm (the patient had a central venous catheter that could not be withdrawn). The outcome of ocular candidiasis was good.

As for outcome, although patients with ocular involvement had a higher mortality rate compared to patients without ocular candidiasis, the difference was not statistically significant (30-day mortality rate38.5% [5/13] vs 18.1% [28/155]; p = 0.13).

### Comparison between patients with and without *Candida* eye involvement

In an attempt to determine whether ophthalmoscopy could be avoided in patients with a low risk of ocular candidiasis, we compared patients with and without ocular candidiasis who had undergone ophthalmoscopy.

As shown in **Table 3**, patients with ocular candidiasis were significantly younger, had more commonly a history of renal disease [53.8% (7/13) vs 26.5% (41/155), p = 0.05] and a higher median Pitt score at presentation of the candidemia [3 vs 1 p = 0.01]. Fungemia causing ocular candidiasis was more frequently persistent [61.5% (8/13) vs 26.5% (41/155), p = 0.02], was produced by *C*. *albicans* [76.9% (10/13) vs 47.1% (73/155), p = 0.04], spread to other organs [38.5% (5/13) vs 9.7% (15/155), p = 0.01], and conditioned more need for hemodialysis [30.8% (4/13) vs 1.9% (3/155), p = 0.001]. As for antifungal treatment, patients with ocular candidiasis more commonly received an adequate antifungal therapy within the first 48 hours [100 (13/13) vs 67.7% (105/155), p = 0.01], and had a longer length of antifungal therapy (mean days 22.2 vs 39.6, p = 0.047). No further differences were found between the groups regarding demographics, risk factors, and management of candidemia. In the multivariate analysis **(Table 4)**, the independent risk conditions for eye involvement were need for hemodialysis after candidemia (OR, 19.4; 95% CI, 1.7–218.4) and involvement of organs other than the eye (OR, 5.4; 95% CI, 1.1–25.7).

**Table 3:** Univariate analysis of risk factors for ocular candidiasis.

Variable	No eye involvement (n = 155)	Eye involvement (n = 13)	*p*
**Age, years, (mean ± SD)**	64.6 ± 14.7	54.5 ± 16.4	**0.02**
**Sex, male (%)**	84 (54.2)	10 (76.9)	0.14
**Hospitalization**			
Medical ward	58 (37.4)	2 (15.4)	0.13
ICU setting	43 (27.7)	5 (38.5)	0.52
Surgical ward	42 (27.1)	5 (38.5)	0.35
Emergency department	8 (9.5)	1 (2.5)	0.57
Others	4 (2.6)	0 (0)	1
**Type of infection**			
Nosocomial	141 (91.0)	11 (84.6)	0.35
Community-acquired	13 (8.4)	2 (15.4)	0.32
Health-care–associated	1 (0.6)	0 (0)	1
**Time between hospitalization and onset of candidemia**	22.0 (13–35)	21.5 (14–34)	0.65
**Hospitalization within previous 3 months**	100 (64.5)	8 (61.5)	1
**Underlying conditions**			
Cancer	67 (43.2)	9 (69.2)	0.08
Solid tumor	56 (36.1)	8 (61.5)	0.08
Cardiovascular disease	53 (34.2)	5 (38.5)	0.76
Renal failure	41 (26.5)	7 (53.8)	0.05
Diabetes mellitus	39 (25.2)	3 (23.1)	1
Neurologic disease	35 (22.6)	3 (23.1)	1
Surgery (all types <30 days)	24 (15.5)	4 (30.8)	0.23
Liver disease	22 (14.2)	1 (7.7)	1
Transplant recipients	15 (9.7)	1 (7.7)	1
Neutropenia	12 (7.7)	2 (15.4)	0.29
Leukemia	9 (5.8)	1 (7.7)	0.56
Mucositis	8 (5.2)	2 (15.4)	0.17
Autoimmune disease	7 (4.5)	1 (7.7)	0.48
Lymphoma	2 (1.3)	0 (0)	1
HIV/AIDS	2 (1.3)	0 (0)	1
**Pitt score**	1 (0–2)	3 (0.5–4)	**0.01**
**Risk factors**			
Antibiotic therapy within previous 30 days	147 (94.8)	12 (92.3)	0.52
Immunosuppressive therapy	52 (33.5)	6 (46.2)	0.37
Antifungal therapy within previous 30 days	43 (27.7)	1 (7.7)	0.18
Corticosteroids at the time of candidemia	31 (20.0)	4 (30.4)	0.47
**Clinical picture**			
Sepsis	126 (81.3)	9 (69.2)	0.28
Severe sepsis	16 (10.3)	3 (23.1)	0.17
Septic shock	13 (8.4)	1 (7.7)	0.52
**Origin**			
Catheter	83 (53.5)	5 (38.5)	0.39
Primary	72 (46.5)	8 (61.5)	0.39
Urinary tract	13 (8.4)	0 (0)	0.6
Intra-abdominal	5 (3.2)	0 (0)	1
***Candida* species**			
*C.albicans*	73 (47.1)	10 (76.9)	0.04
*C. parapsilosis*	34 (21.9)	2 (15.4)	0.73
*C. glabrata*	20 (12.9)	0 (0)	0.36
*C. tropicalis*	17 (11.0)	0 (0)	0.36
*C. krusei*	4 (2.6)	0 (0)	1
Others	28 (18.1)	1 (7.7)	0.47
**Intravascular catheter at the time of candidemia**			
Overall	151 (97.4)	13 (100)	1
Central	122 (78.7)	12 (92.3)	0.47
Peripheral	73 (47.1)	4 (30.8)	0.39
**Type of catheter**			
Subclavian	40 (25.8)	5 (38.5)	0.33
Jugular	35 (22.6)	5 (38.5)	0.19
Peripherally inserted central catheter	29 (18.7)	1 (7.7)	0.47
Arterial	20 (12.9)	3 (23.1)	0.39
Tunneled central venous catheter	18 (11.6)	1 (7.7)	1
Femoral	17 (11.0)	2 (15.4)	0.64
**Catheter removal**	135 (87.1)	11 (84.6)	0.68
**TPN during candidemia**	78 (50.3)	7 (53.8)	1
**Complications due to candidemia**			
Other organs involvement	15 (9.7)	5 (38.5)	**0.01**
ICU admission	9 (5.8)	1 (7.7)	0.56
Dialysis	3 (1.9)	4 (30.8)	**0.001**
**Concomitant bacterial infection**	19 (12.3)	1 (7.7)	1
**Initiation of antifungal therapy**			
< 24 h since positive BC	59 (38.1)	6 (46.2)	0.56
< 48 h since positive BC	105 (67.7)	13 (100)	0.01
< 72 h since positive BC	125 (80.6)	13 (100)	0.12
**Time to initiation of antifungal therapy since positive BC, median, days (IQR)**	2 (1–3)	2 (1–2)	0.43
**First antifungal therapy**			
Azoles	88 (56.5)	10 (76.9)	0.24
Echinocandins	53 (34.2)	3 (23.1)	0.54
L-AMB	14 (9.0)	0 (0)	0.60
**Length of antifungal therapy**	22.2 ± 14.5	39.6± 36.6	**0.047**
**Persistent candidemia**	41 (26.5)	8 (61.5)	**0.02**
**Length of antifungal treatment (median, days)**	3 (1–15)	2 (1–16)	0.94
**Death**			
7-day mortality	6 (3.9)	0 (0)	1
Overall mortality	28 (18.1)	5 (38.5)	0.13

**BC** blood culture; **ICU** intensive care unit; **IQR** interquartile range; **L-AMB** liposomal amphotericin B; **TPN** total parenteral nutrition.

**Table 4:** Risk conditions for ocular candidiasis. Multivariate analysis.

VARIABLES	Odds ratio	95% confidence interval	*p*
**Dialysis after candidemia**	**19.4**	**1.7–218.4**	**0.02**
**Other organs involvement**	**5.4**	**1.1–25.7**	**0.04**
Fungemia due to *C*. *albicans*	4.2	0.8–20.2	0.08
Persistent candidemia	3.0	0.7–13.7	0.15
Initial echinocandin therapy	2.6	0.4–16.3	0.29
Pitt score	1.2	0.8–1.6	0.40

## Discussion

Our study shows that, despite the recommendation by several guidelines that systematic ophthalmoscopy should be performed in all patients with candidemia [6–9], as many as 50% of patients never actually underwent ophthalmoscopy. Furthermore, the yield of this examination is low, and ocular infection is uncommon and mostly asymptomatic. Our data also show no independent impact of ophthalmoscopy on the outcome of candidemic patients.

The incidence of ocular candidiasis in patients with candidemia varies from 50% in older studies [14–17] to less than 5% in more recent ones [18, 19]. In the present report, the overall incidence of ocular candidiasis was 7.7%, which is consistent with that observed by Shah et al [20], who found a 7.9% frequency of chorioretinitis, with no cases of endophthalmitis. The widespread use of early antifungal therapy and improvements in diagnosis may explain the lower incidence of ocular candidiasis observed in recent years [18, 19, 21].

Nevertheless, recent guidelines recommend performing ophthalmoscopy in all candidemic patients [6–9], stating that “missing and not appropriately treating *Candida* endophthalmitis could have great consequences for the patients” [9]. However, the quality of the evidence supporting this recommendation is low, as it is based on the clinical judgment of the Expert Panel members.

This weakness in the recommendations provided by guidelines is reflected in the wide range of rates of ophthalmoscopy in candidemic patients (53% to 75%) reported in the medical literature [22, 23]. In the same sense, although all patients included in the CANDIPOP study were prospectively followed by infectious disease specialists, we found that in “real life”, ophthalmoscopy was performed in less than 50% of the study population. In our fully publicly funded health care system, this low percentage cannot be attributed to cost- or reimbursement-related factors.

As for clinical manifestations, we found only 1 patient with ocular symptoms, ie, an incidence of symptomatic disease of 0.27% in the whole candidemic population. Moreover, ocular candidiasis progressed favorably in all except 1 of our evaluable patients, who was asymptomatic and did not experience vision loss. These findings are consistent with those recently reported by Oude Lahof, who observed visual symptoms in only 3.3% of patients with ocular candidiasis and a favorable outcome in almost all evaluable cases [10]. Older studies reporting ocular candidiasis as a "malignant complication" were performed in an era when medical management of candidemia differed substantially from contemporary care [1–5, 24–26]. Furthermore, a high proportion of patients included in such studies had a history of intravenous drug use [5] that was not observed in our series.

Oude Lashof [10] found a similar mortality rate between patients with and without ocular candidiasis (43.3% vs. 36.5%, *p* = 0.31). We also observed nonsignificant differences (*p* = 0.13), thus indicating that the presence of ocular abnormalities does not predict a poor outcome.

Similarly, although the populations undergoing or not undergoing ophthalmoscopy were likely not identical, we did not observe an impact of routine ophthalmoscopy on clinical outcome. Performance of the examination resulted in a change in antifungal therapy in only 5.6% of the 168 patients. On the contrary, ophthalmoscopy generates an increase in hospital costs ($400 per consultation), is uncomfortable for patients, and carries a small risk of acute angle-closure glaucoma [27].

Since ocular candidiasis progressed favorably in almost all patients and given the lack of data on the clinical impact of longer treatment schedules, we believe that the recommendation of systematic dilated ophthalmoscopy for all candidemic patients should be reassessed. An alternative could be a risk-based approach, in which the examination is limited to symptomatic patients, those who do not respond to treatment, or those more likely to acquire ocular candidiasis.

Regarding this aspect, our findings are consistent with those reported by other investigators [10, 15, 20, 28, 29], who found increased severity of the underlying conditions (cancer, need for hemodialysis after candidemia, and corticosteroids) and exposure to a more virulent infection (*C*. *albicans*, persistent fungemia, septic metastasis in other organs) as risk factors for ocular candidiasis.

Our study is subject to a series of limitations. First, the CANDIPOP study was not designed to analyze ocular candidiasis; however, we report the broadest experience to date on ophthalmoscopy in a large population of patients who were prospectively followed by an infectious disease specialist. Second, only 46% of the candidemic patients underwent ophthalmoscopy, with the result that we may have underestimated the involvement of ocular *Candida* infection; however, no clinical manifestations of ocular candidiasis were observed in the group of patients who did not undergo ophthalmoscopy. Finally, no patients with a history of drug addiction were included in the study.

In conclusion, we provide data from a large series of patients with candidemia showing that ophthalmological assessment is frequently omitted and that the rate of ocular candidiasis is relatively low (7.7%), anecdotally symptomatic, and usually associated with a good outcome. A prospective clinical trial evaluating the real benefits of routinely performed ocular assessment in all candidemic patients to limit the use of such a cumbersome, low-yield examination.
